# Rejoice architecture meets social norms to accelerate vaccination in Nepal: Protocol for a mixed-method quasi-experimental study

**DOI:** 10.12688/gatesopenres.13168.1

**Published:** 2020-08-26

**Authors:** Alicia Paul, Kamana Upreti, Shraddha Nepal, Jeevan Lohani, Kriti Adhikari, Rajiv Rimal

**Affiliations:** 1 Department of Health, Behavior and Society, Johns Hopkins Bloomberg School of Public Health, Baltimore, MD, 21205, USA; 2 Nepal Evaluation and Assessment Team, Kathmandu, 44600, Nepal; 3 Nepal Health Research Council, Kathmandu, 44600, Nepal

**Keywords:** social norms, choice architecture, broken windows theory, immunization, study protocol, mixed-methods, quasi-experiment

## Abstract

**Background: **Each year, 600,000 children under 5 years old die from vaccine-preventable diseases globally. Immunization is an effective way to prevent many diseases, saving two to three million lives per year. The Nepal National Government recommends vaccinations for all children for 11 diseases by 15 months of age. However, only 78% of children between 1-2 years of age have received all recommended vaccines and only 43% receive them at the age-appropriate times for which they are scheduled.

**Objectives: **This protocol describes the development of an intervention – called “Rejoice Architecture” – that is informed by three theoretical perspectives: choice architecture, the broken windows theory, and the theory of normative social behavior. We also describe a mixed-methods approach to develop the intervention, which will improve the physical and social environments of health facilities in Makwanpur, Nepal. We hypothesize this intervention will improve immunization behaviors and intentions among mothers of children younger than 2 years, pregnant women, and prospective mothers.

**Methods: **We describe the qualitative formative assessment to understand existing attitudes, norms, and behaviors among caregivers, healthcare workers, and government representatives. The formative assessment will include in-depth interviews, key informant interviews, and focus group discussions. We also describe the overall quasi-experimental study design, used to assess intervention impact.

**Impact: **This study will contribute to the social and behavioral change communication intervention research by offering a novel strategy for increasing immunization. This study will also illustrate to policymakers the value of structural change for health service delivery.

## Introduction

 Communicable diseases constitute a global health threat, with low- and middle-income countries experiencing the greatest burden ( The World Medical Association, 2020). Each year, 1.5 million people die from vaccine-preventable diseases, 600,000 of whom are children under the age of 5 ( Gavi, 2020). Immunization is an effective way to prevent disease and save lives, with two to three million lives saved by vaccines every year ( World Health Organization, 2019). Individuals benefit from immunization through illness prevention, reduced healthcare costs, and greater productivity for themselves and their caregivers ( Gavi, 2020; Njau & Cairns, 2016; Ozawa *et al.,* 2012). Children, who are often at greater risk of morbidity and mortality from communicable disease, experience greater cognitive ability, physical strength, and school performance when they receive vaccines ( Gavi, 2020; Nandi & Shet, 2020). Immunizations also contribute to improved community health through herd immunity and greater economic stability from long-term cost savings ( Fox *et al.,* 1971; Gavi, 2020; Ozawa *et al.,* 2012; Ozawa *et al.,* 2017). 

Currently, 20 million children worldwide have not received the minimum basic vaccines they need to live long, healthy lives. Furthermore, only 11% have received the full dosage recommended by the World Health Organization ( Gavi, 2020). This coverage gap is present in Nepal, where only 78% of children between 1–2 years have received all recommended vaccines ( Ministry of Health Nepal *et al.,* 2017). It is important to note, though, that while the majority of children receive their vaccines by the age of 2, only 43% receive them at the age-appropriate times for which they are scheduled ( Ministry of Health Nepal *et al.,* 2017). Moreover, immunization coverage is highly variable in Nepal, with rates ranging from 65% in some provinces to 93% in others ( Ministry of Health Nepal *et al.,* 2017). Coverage is dependent on the specific vaccine as well, ranging from 73% for the first dose of the Pneumococcal vaccine to 98% for the Bacille Calmette Guerin (BCG) vaccine ( Ministry of Health Nepal *et al.,* 2017). These data demonstrate a critical need to ensure consistent and comprehensive coverage of immunization, including in Nepal.

### National Immunization Programme

Since its inception in 1979, Nepal’s National Immunization Programme (NIP) has been one of the government’s highest priorities, aiming to “reduce child mortality, morbidity and disability associated with vaccine preventable disease” ( Ministry of Health Nepal, 2017, n.p.). The program, which originally offered only BCG and Diphtheria Pertussis Tetanus (DPT) vaccines in three districts, expanded in 1988 to cover all 75 districts in the country and also provide oral polio and measles vaccines ( Department of Health Services, 2018). As of 2020, seven additional vaccines (hepatitis B, Hemophilus influenza b, Japanese encephalitis, injectable polio vaccine, pneumococcal conjugated vaccine, Rota vaccine and rubella) have been added to the program, providing vaccines for 11 vaccine-preventable diseases in total ( Ministry of Health and Population Nepal, 2020).

Immunization services are primarily delivered through government networks, such as health facilities, outreach clinics, and mobile clinics, but an upward trend of delivery by private institutions is being observed in urban areas ( Child Health Division, 2011). Vaccines and related logistics are provided free of cost to all health facilities, both public and private, by the government ( Child Health Division, 2011; Department of Health Services, 2018). Many health facilities elect to administer vaccines on one pre-specified day per month, called “Immunization Day.” This system helps providers track vaccine uptake in their communities by reaching all children in need of vaccines on the same day, and it also helps parents remember to bring their child for their next immunization.

The NIP played a major role in meeting the fourth target of the Millennium Development Goals to reduce the number of under-five deaths to less than 40 per 1,000 live births ( Ministry of Health and Population Nepal, 2020; National Planning Commission, 2017). The country achieved a number of successes, including being declared polio free in 2014, maintaining elimination status of maternal and neonatal tetanus since 2005, controlling rubella and cognitive rubella syndrome in 2018, and progressing towards the elimination of measles ( Department of Health Services, 2018; Ministry of Health and Population Nepal, 2020). More recently, various innovative measures have been taken to declare full immunization in the country. One such initiative, entitled “Reaching Every Child,” was implemented in 2012 and has seen success by providing greater ownership to local political bodies. In total, 56 of 77 districts have declared full immunization; however, 14 districts continue to show dropout rates greater than 10% and 26 districts, including the capital city Kathmandu, have coverage below 80% ( Department of Health Services, 2018).

### Facilitators and barriers of immunization

Studies in Nepal show that institutional delivery is a prominent facilitator of vaccine uptake ( Acharya *et al.,* 2019; Shrestha *et al.,* 2016). Shrestha *et al.* (2016) offer one potential reason for this, stating that the first vaccine (BCG) is often administered immediately after birth, which is more likely to be readily available in a health facility setting than at home. Furthermore, in institutions, new mothers are surrounded by numerous healthcare staff who can share their recommendations for future vaccines ( Shrestha *et al.,* 2016).

Multiple studies in Asia and Africa have found a positive association between parental knowledge of immunization and vaccine uptake ( Odusanya *et al.,* 2008; Owino *et al.,* 2009; Perry *et al.,* 2020). This can refer to knowledge about the purpose of vaccines, appropriate ages to receive specific vaccines, age at which a child should complete the vaccines, and symptoms of vaccines, among other topics ( Odusanya *et al.,* 2008). Some studies show that even in areas of low literacy, parental knowledge is a significant determinant of full immunization ( Matsumura *et al.,* 2005; Odusanya *et al.,* 2008). An important aspect of parental knowledge is awareness of the vaccine schedule, which is also strongly correlated with immunization coverage ( Owino *et al.,* 2009; Shrestha *et al.,* 2016). In Nepal, full immunization requires seven separate visits over 15 months; it is essential that parents know and remember this schedule in order to bring their children to the facility at the appropriate time ( Acharya *et al.,* 2019).

For countries like Nepal that rely on immunization cards to track children’s vaccine records, immunization card retention is a major facilitator of uptake ( Acharya *et al.,* 2019; Perry *et al.,* 2020). Perry *et al.* (2020) found that in Bangladesh where, like Nepal, immunization cards are required to receive vaccines, parents are met with anger, shouting, and in some cases a fee when they lose or forget their card. Improving immunization card retention has serious implications for encouraging immunization in Nepal, where the retention rate for immunization cards is only 52% ( Acharya *et al.,* 2019).

Barriers to vaccine uptake exist at the individual and health system levels. Several studies suggest an association between parents’ negative attitudes toward immunization and vaccine incompletion. Two studies in Nepal point to a fear among parents associated with vaccinating their children, particularly in instances when the child is perceived as being “too ill” to be administered a vaccine ( Basel & Shrestha, 2012; Shrestha *et al.,* 2016). Across various countries in Asia and Africa, the most common concern among parents is the potential for side effects and the appropriate response, the fear of which has caused parents to refuse vaccines for their child ( Favin *et al.,* 2012; Owino *et al.,* 2009; Perry *et al.,* 2020). To compound the issue, negative attitudes may also be strengthened by competing priorities ( Favin *et al.,* 2012; Shrestha *et al.,* 2016; Vonasek *et al.,* 2016). Because immunization can sometimes require long distance travel, followed by long wait times, parents in these circumstances must weigh the benefits of vaccines against the costs of foregoing responsibilities like working, caring for other children, or completing household chores ( Favin *et al.,* 2012).

A systematic review of incomplete vaccinations in low- and middle-income countries found that a significant factor for under-vaccination could be explained by health system issues, such as access to services, inadequate health worker knowledge, and vaccine shortage ( Rainey *et al.,* 2011). These findings were further established by Favin *et al.* (2012), who found in their review frequent instances of unreliable services (e.g., appointment cancelations, absent providers, lack of supplies) and disrespectful staff (e.g., screaming at mothers, discouraging vaccination). What is less known, and what this project will seek to understand, is the influence of health facilities’ physical infrastructure on vaccine uptake and the underlying conceptual mechanisms that explain this relationship with facility-level factors.

### Conceptual framework

This project is informed by ideas from three theoretical frameworks: choice architecture, the broken windows theory, and the theory of normative social behavior. Choice architecture ( Thaler & Sunstein, 2008) is based on the idea that human behavior, to some extent, is driven by expediency in decision-making – that people do not want to expend a lot of effort thinking critically about the pros and cons of decisions they need to make. If the decision-making environment is configured in a certain way to promote a particular decision, many will opt for that decision ( Thaler *et al.,* 2014). In explaining this theory, the authors write “For reasons of laziness, fear, and distraction, many people will take whatever option requires the least effort, or the path of least resistance” (p. 430). At its most basic level, choice architecture involves two components – options available to the decision-maker, including the number of options from which to make a decision, and the manner in which the options are framed ( Johnson *et al.,* 2012). 

The overall idea here is that people’s behaviors can be changed by configuring the environment so that the default choice is in line with the desired behavior. Thus, structurally, if the stairs in a building are the first point of access and taking the elevator requires walking to the back of the building, people are more likely to take the stairs. Applied to the vaccination context, choice architecture principles could be used to immunize an institutionally delivered child by default, but allowing parents to opt out if they so choose (as opposed to making the immunization be the opt-in choice).

While choice architecture focuses on optimal structuring of the environment, the broken windows theory ( Wilson & Kelling, 1982) asserts that the condition of disrepair in the environment (e.g., preponderance of broken windows) signals to people that social order is in decay. This further communicates to people that they will not be punished if they, too, engage in behaviors that, literally and metaphorically, break more windows. Hence, the state of broken windows in a community further perpetuates social disorder. In this project, we ask if the reverse is also true in an immunization context: if the physical and social environment in which vaccination is being delivered is improved – from a state of disrepair and disrespect to a state of rejuvenation and respect – will more caregivers come for vaccinations?

One of the mechanisms underlying the broken windows theory is normative influence, the idea that people’s behaviors are driven by their beliefs about what others are doing (called descriptive norms) and pressures they experience to conform (injunctive norms; Cialdini *et al.,* 1990). When people perceive that an environment is in disrepair, they also perceive that others do not care and, by extension, that it would be acceptable for they themselves not to care. In this way, broken windows influence social norms, which in turn affect behavioral choices. The relationship between normative beliefs and behaviors is addressed by the theory of normative social behavior ( Rimal & Real, 2005), which posits that social norms affect behaviors not only directly, but also indirectly when other factors are present. For example, social normative influences are heightened when people have high self-efficacy ( Jain & Humienny, 2020; Jang *et al.,* 2013; Johnson, 2011) to enact the behavior and when they perceive that the behavior confers many benefits ( Lapinski *et al.,* 2014; Rimal, 2008).

### Objectives

This protocol seeks to meet three key objectives:

1. Describe the process by which the formative assessment will be conducted to understand existing attitudes, norms, and behaviors relating to immunization.
2. Describe how the results of the formative assessment will be translated to improve the intervention design.
3. Describe the methodology that will be used to evaluate the impact of the intervention on immunization behavior and intentions.

## Methods

### Study setting

**Figure 1.**
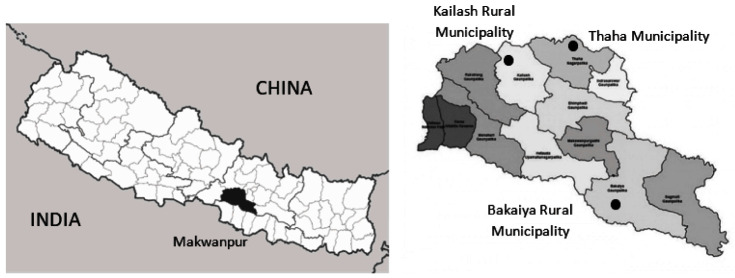
Geographical location of study.

The study is being conducted in three Palikas (equivalent to a municipality) of the Makwanpur District of Nepal: Thaha Municipality, Kailash Rural Municipality and Bakaiya Rural Municipality (see Figure 1). As per the NIP schedule, the rate of immunization coverage of children under one year in Makwanpur is 76.7% ( Ministry of Health Nepal Department of Health Services, 2018). Furthermore, Makwanpur is accessible from Kathmandu, the capital of Nepal, is neither in the lowlands (Terai) or mountainous regions, and is large in size. These features make Makwanpur an ideal candidate setting to maximize feasibility and external validity as well as minimize contamination.

In each of the three Palikas, we selected one ward (the administrative unit below a Palika) as a treatment site, and chose a clinic in the ward based on our desire to maximize geographic diversity across the three Palikas. For each ward in the treatment arm, we selected another ward and clinic as a treatment site in that Palika, matched on population characteristics with the treatment ward. Each control facility is located in a separate geographic vicinity from the treatment facility in its Palika to minimize contamination.

### Study team

This research is a partnership between Johns Hopkins University (JHU) and Nepal Evaluation and Assessment Team (NEAT), a nongovernment organization in Kathmandu, Nepal. The research team includes university-based researchers with expertise in social norms, social and behavioral change communication interventions, and mixed-methods research in global health contexts.

### Research design and intervention

**Figure 2.**
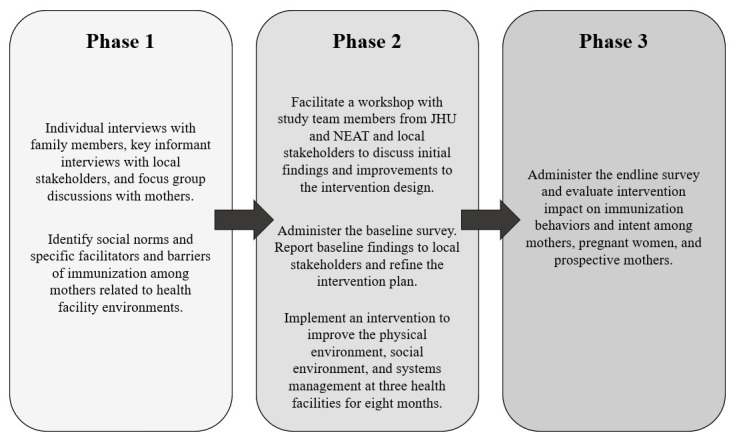
Research study workflow.

This study is composed of three phases: 1) qualitative formative assessment, 2) intervention implementation, and 3) quantitative impact assessment (see Figure 2). Mixed methods approaches can be useful for producing a more comprehensive picture of the issue at hand and building on initial findings ( Denscombe, 2008). For this study, the formative assessment will be used to understand individual and group perspectives of immunization practices in their communities and opinions about their local health facilities. The findings from this assessment will be used to build on the Rejoice Architecture intervention plan, which will include improvements to the health facility through three basic components: 1) physical environment and infrastructure, 2) social environment and communication with health staff, and 3) systems management and scheduling. Specific intervention activities will include painting walls, providing furniture, adding greenery, designing a client-provider communication checklist, and implementing an appointment reminder system, among others. Activities will be refined, expanded upon, or added based on priorities identified in the formative assessment. Following this assessment, a clustered quasi-experimental survey design will be utilized to determine overall impact of the intervention.

## Qualitative methods

The formative assessment will be used to understand the attitudes of and behaviors toward immunization within each community and show how mothers, other community members, and community leaders perceive the environment of their local health facilities. This assessment will also aid us in identifying mothers’ priorities for making the health facilities’ environments more welcoming to patients and caregivers.

### Study design

We plan to collect qualitative data from five of the six study sites, excluding one control site. Data will be collected primarily from treatment sites to ensure the needs of the treatment sites are identified for the intervention design. Qualitative data will include approximately 10 in-depth interviews, 13 key informant interviews, and eight focus group discussions. Refer to Table 1 for our target breakdown of qualitative data by treatment and control sites. It is important to note that due to travel restrictions to prevent spread of COVID-19, actual numbers may vary.

**Table 1.** Breakdown of qualitative data collection by treatment and control facilities.

Participant	Session Type	# Sessions (Control)	# Sessions (Treatment)	Total
Mothers	FGD	2	6	8
Fathers	IDI	2	3	5
Grandmothers	IDI	2	3	5
Health worker	KII	1	3	4
Government representative	KII	2	3	5
FCHV	KII	1	3	4

Furthermore, we will match the interviewer and interviewee by gender when possible to strengthen rapport and help the participant feel more at ease. Researchers will conduct all assessments in Nepali, the local language, and collect audio recordings, permitting consent.

### Participants

We will conduct in-depth interviews with fathers and grandmothers of children under the age of 2. We will conduct key informant interviews with Female Community Health Volunteers (FCHVs); health workers, with priority given to workers in a leadership role (i.e. Health In-Charge, Immunization Program Manager, etc.); and representatives of the local government (i.e. Ward Chair, chair of the Health Facility Operation Management Committee, etc.). Focus group discussions will consist of mothers of children under 2 years of age. All participants will be at least 18 years or older and live within a one-kilometer radius of a study health facility or work for, oversee, or volunteer at a study health facility.

### Sampling

FCHVs will provide assistance in identifying eligible participants for the interviews. They maintain an updated list of community members served by their health facilities, including families with newborn children and record of the child’s immunization status. Using these lists, FCHVs will identify homes with potential participants for us to contact and recruit. For two of the eight focus groups, we will only recruit individuals from marginalized communities based on their caste to broaden our scope and gain a more comprehensive image of the community and their array of experiences. Each discussion group will consist of five to nine participants. For In-depth interviews, potential participants will be excluded if any member of their family has participated in one of our interviews or focus groups. Participants of the key informant interviews will be identified through discussions with community leadership.

### Instruments

We have prepared the qualitative instruments using influence from the theory of normative social behavior ( Rimal & Real, 2005) and choice architecture ( Thaler *et al.,* 2014). Interview and focus group instruments also include concepts from the literature on facilitators and barriers of immunization ( Acharya *et al.,* 2019; Shrestha *et al.,* 2016) and adapted survey questions regarding clinic infrastructure from the Rapid Assessment Tool ( Scholz *et al.,* 2015) and the Site Assessment for Maternal and Newborn Health Programs ( JHPIEGO, 2004). The instruments were adapted for local use based on feedback from the Nepali team on cultural considerations including language, phrasing, and use of probes. The tools will undergo pre-testing and will be revised to ensure smooth flow of questions, avoid confusing technical terms and jargon, and assure appropriate phrasing to avoid misunderstanding or offense.

Topics covered in each interview and focus group will vary based on the instrument in use as well as flow of the conversation. All qualitative instruments include questions regarding facilitators, barriers, and social norms of immunization. The focus group guide will also cover the physical and social characteristics that mothers desire in an ideal health facility, a description of their current health facility, and typical communication between caregivers and health care providers. The in-depth interview guide will cover the family’s personal experiences with vaccination, family involvement in child health and decision-making, and aspirations for the child’s education and career. The key informant interview guide will include items regarding immunization coverage in the ward, typical communication between caregivers and health care providers, a physical description of the local health facility, opinions of their workload, and feasibility of the proposed intervention. Interview guides are available as *Extended data* ( Paul, 2020).

## Quantitative methods

Whereas the qualitative component of the research helps inform the intervention design and the quantitative instrument, the quantitative component’s primary use is in tabulating the impact of the intervention in as rigorous a manner as possible.

### Study design

We will use a clustered quasi-experimental design with panel data to measure the impact of the intervention on immunization behaviors, intent to vaccinate, and uptake of other health services. Participants from each of the six study sites will complete a baseline survey preceding intervention implementation and an end-line survey approximately eight months later. Data collectors will read questions aloud to participants and then record responses on a handheld tablet. Surveys are available as *Extended data* ( Paul, 2020).

In addition, the physical conditions of the health facilities and immunization centers will be assessed using checklists at baseline. These checklists will be used to corroborate subjective reports from interviews, focus group discussions, and surveys and assist with identifying needed resources to include in the intervention design. Our data collectors will visit each health facility and the surrounding immunization centers on the designated “Immunization Day” to complete the checklists.

### Participants

Eligible participants include mothers of children younger than two years, pregnant women, and prospective mothers. Prospective mothers are defined as women of reproductive age (18–30 years) who are not currently pregnant and do not have a child two years or younger, but express interest in having a child in the future. All participants must be 18 years or older and live within a one-kilometer radius of the health facility being studied. Potential participants will be excluded if they or a member of their family participated in any of the qualitative assessments.

### Sampling

Like the qualitative methods, FCHVs from each ward will assist in identifying women in the community who fit the eligibility requirements. With their list of names and locations, data collectors will randomly select houses for recruitment. The target sample size is *n* = 950 (treatment = 475, control = 475) with an anticipated attrition rate of 20%. This was determined by assuming an increase in vaccination rates from 83% (current) to 93% (envisioned), an alpha level of 0.05 and power of 80%, and data collection from 6 clusters. We estimate an intraclass correlation of 0.01, making the required sample size 380 in the treatment and 380 in the control groups. However, because this is a panel design, we envision an attrition rate of 20%, which brings our sample size at baseline to 950. We will sample four cohorts of women with children younger than one year, women with children between 1–2 years, women who are pregnant at baseline, and prospective mothers. We anticipate greatest availability of women in the first two cohorts, and the least availability of pregnant women, which we expect will create variation in our sample sizes between cohorts. For the complete anticipated sampling breakdown, see Table 2.

**Table 2.** Breakdown of quantitative data collection by treatment and control facilities.

Participant	Control	Treatment	Total
Women with children <1 year	150	150	300
Women with children 1–2 years	150	150	300
Pregnant women	50	50	100
Prospective mothers	125	125	250
Total	475	475	950

### Instruments

Baseline and end-line surveys will measure primary outcomes of immunization behavior and intent to immunize future children. A secondary outcome of general service utilization will also be measured. To assess influencing variables, surveys will measure vaccine knowledge, vaccine attitudes and beliefs, self-efficacy, information sources, and perceived social norms regarding immunization. To understand participants’ experiences and perceptions of their health facilities, surveys will measure quality of communication with providers, past experiences with pregnancy and delivery, and perceptions of their facility’s physical environment. Lastly, the surveys will measure concepts relating to the individual, including their demographics, common methods of communication, interspousal relationships (if married), and mental health. End-line surveys will include all items from the baseline surveys plus an additional section to measure exposure to the intervention.

Surveys will vary slightly depending on the participant’s classification. For instance, mothers of children younger than 2 will receive a survey which includes questions about their immunization behaviors. Conversely, pregnant women and prospective mothers will receive a similar survey that, instead, includes questions about their intentions to immunize their future children and, for some items, requests they answer as if they currently have a child younger than 2. All other survey items will be identical for participants, regardless of their classification.

The Health Facility Checklist will measure the quality of the interior physical environment, exterior physical environment, consultation room, storage and supplies, and accessibility of health facilities. Immunization centers are significantly smaller than health facilities and are only used once per month to provide vaccines. Therefore, the Immunization Center Checklist has been modified to measure the same concepts with only the relevant items. Both checklists use items adapted from the Rapid Assessment Tool ( Scholz *et al.,* 2015) and the Site Assessment for Inpatient Postpartum Care ( JHPIEGO, 2004).

### Outcomes

***Immunization behavior and intent.*** Items measuring immunization behavior and intent are adapted from the Nepal Woman Questionnaire from the 2016 Demographic and Health Survey (DHS) and reflect either the degree to which participants have followed the National Immunization Schedule of Nepal (for mothers) or their intent to follow the schedule in the future (for pregnant women and prospective mothers; Ministry of Health and Population, 2020; Ministry of Health Nepal *et al.,* 2017).

***General service use.*** Items measuring health service utilization will address how often the participant has visited their health facility in the last three months for common health issues including family planning, pregnancy checkups, Tuberculosis treatment, diarrhea treatment, pneumonia treatment, and child sick visits.

## Data collection

### Training

An in-person training will be held in Kathmandu, Nepal for all data collectors to prepare for the qualitative and baseline assessments. The Principal Investigator will conduct the training alongside research staff from the data collection agency. The training will be held for five days with an objective to prepare the researchers for qualitative and quantitative data collection. Training topics will include key concepts of the study like child health and immunization, techniques for probing in interviews and facilitating focus group discussions, and ethical research conduct. 

Following researcher training, we will pretest all instruments and research methods in one ward in Makwanpur that is not part of the actual study. During pretesting, supervisors will observe and provide feedback on research techniques.

### Instrument development

All instruments were designed through an iterative process in which team members from the U.S. and Nepal collaborated to provide feedback and make revisions. Each instrument underwent multiple rounds of revisions until all team members concluded the items appropriately reflected the study’s objectives, well-developed theory and literature, and cultural context. Each instrument was translated from English to Nepali by a team member to ensure that questions would be understandable to participants without compromising construct validity.

All instruments will undergo a final round of revisions during the pre-testing phase during which time we will assess the instruments for flow, cultural appropriateness, and use of technical terms and jargon. Data collectors will provide feedback from their experiences which will be incorporated into final revisions for the instruments.

### Consent process

Prior to collection of any data, participants will be read aloud and provided an information sheet detailing the purpose of the study, expected duration of participation, types of questions they may be asked, and their options to decline to participate or quit at any time. Consent will be received immediately following recruitment at the individual’s home or place of work in a private space. Consent will be received with a written signature. Informed consent materials are available as *Extended data* ( Paul, 2020).

## Data analysis

### Qualitative

As data are being collected, researchers will record field notes to capture the overall picture of the interview or focus group. They will note their perceptions of the participant’s attitude and willingness to engage, as well as the surroundings, interruptions, or other notable characteristics of the session. Following data collection, team members will transcribe the recordings and translate them from Nepali to English.

Following Braun & Clarke’s (2006) guidelines, we will adopt an iterative, thematic analysis approach to analyze the in-depth interviews, key informant interviews, and focus group discussions to uncover prominent themes. This approach will allow us to thematize both the supply-side (e.g., clinic environments, providers’ communication patterns) and demand-side (e.g., attitudes, physical access, etc.) facilitators and barriers of immunization behaviors ( Braun & Clarke, 2006).

Researchers with experience in qualitative analysis will independently read through transcripts to gain familiarity with the data and develop an initial codebook. The team will then code transcripts independently, using QSR International's NVivo software, meeting regularly to revise the codebook as themes emerge. Codes will be added both deductively, based on our research question, and inductively as new trends appear. Team members will each be paired with another teammate, rotating on a weekly schedule, to compare codes, discuss discrepancies, and reach consensus. All transcripts will be coded by at least two team members. We will determine prominent themes by comparing codes across the in-depth interviews, key informant interviews, and focus group discussions; running word queries; and creating visual conceptual maps to link patterns.

### Quantitative

The primary objective of the quantitative analysis is to determine whether the Rejoice Architecture intervention results in greater vaccination uptake (or intentions for vaccination) among mothers (or prospective mothers) in the treatment, compared to the control, arm. Hierarchical linear regressions (HLM) will be used to test the primary hypothesis that vaccination rates (number of newborns vaccinated divided by total live births within a catchment area) in the treatment group will be significantly greater (goal is 10 percent) than that in the control group. This analysis will take into account the clustered nature of data within each of the six catchment areas (three in treatment and three in control areas).

### Limitations

This study may be limited in several ways. For instance, the study sites were selected purposively and are in one of 77 districts in Nepal; therefore, generalizability could be limited. Findings may also be limited to rural settings among certain ethnic groups in Makwanpur and may vary among other geographic or ethnic contexts.

Considering the current COVID-19 pandemic, intervention implementation may be delayed, and data will likely reflect the pandemic’s effects on facility closures, attitudes toward help-seeking, and health-related behaviors. However, both treatment and control facilities will be exposed to the virus and Nepal’s national response, and so no significant differences due to COVID-19 are expected between groups.

### Contribution to the field

Currently in phase 2 of the study, we have collected baseline data and modified the Rejoice Architecture intervention design, and will implement the intervention when COVID-19 travel restrictions are lifted. We expect this project will make incremental, though important contributions to the global efforts in increasing immunization and reducing child mortality. The iterative and multi-phase procedure will allow for constant adaptation of the intervention, driven by a deepened understanding of individual and contextual factors that will be achieved through community-based participation at every phase. This theory-driven approach will offer a novel connection between community-level theory and individual behavior, bridged by changes in social norms. More specifically, this study will test the pathway between physical infrastructure, mothers’ behavior and child immunization, and will therefore provide direction for future research aiming to address immunization uptake and other service use in LMICs.

### Ethics and dissemination

The Institutional Review Boards with Johns Hopkins University and the Nepal Health Research Council approved this study. All participants will provide written consent before data collection. Findings from this study will be disseminated in participating Palikas in Makwanpur, at international research conferences, and through peer-reviewed journals.

## Data availability

### Underlying data

No underlying data are associated with this article.

### Extended data

Open Science Framework: Rejoice Architecture Meets Social Norms to Accelerate Vaccination in Nepal: Protocol for a Mixed-Method Quasi-Experimental Study. https://doi.org/10.17605/OSF.IO/JYBK4 ( Paul, 2020).

This project contains the following extended data:

- Interview and focus group guides
- Baseline survey instruments
- Consent forms

Extended data are available under the terms of the Creative Commons Zero "No rights reserved" data waiver (CC0 1.0 Public domain dedication).

## References

[ref-1] Acharya K Lacoul M Bietsch K: Factors Affecting Vaccination Coverage and Retention of Vaccination Cards in Nepal. DHS Further Analysis Reports No. 121. Rockville, Maryland, USA: ICF.2019. Reference Source

[ref-2] Basel PL Shrestha IB: Factors associated with dropout between Bacille Calmette Guerin (BCG) and measles vaccination in a village development committee of a district. *J Nepal Health Res Counc.* 2012;10(21):147–151. 23034378

[ref-3] Braun V Clarke V: Using thematic analysis in psychology. *Qual Res Psychol.* 2006;3(2):77–101. 10.1191/1478088706qp063oa

[ref-4] Child Health Division: National immunization program: Reaching every child, Comprehensive multi-year plan 2068-2072. Government of Nepal.2011. Reference Source

[ref-5] Cialdini RB Reno RR Kallgren CA: A focus theory of normative conduct: Recycling the concept of norms to reduce littering in public places. *J Pers Soc Psychol.* 1990;58(6):1015–1026. 10.1037/0022-3514.58.6.1015

[ref-6] Denscombe M: Communities of practice: A research paradigm for the mixed methods approach. *J Mix Method Res.* 2008;2(3):270–283. 10.1177/1558689808316807

[ref-7] Department of Health Services: Annual report 2074-75. Government of Nepal.2018. Reference Source

[ref-8] Favin M Steinglass R Fields R: Why children are not vaccinated: A review of the grey literature. *Int Health.* 2012;4(4):229–238. 10.1016/j.inhe.2012.07.004 24029668

[ref-9] Fox JP Elveback L Scott W: HERD IMMUNITY: BASIC CONCEPT AND RELEVANCE TO PUBLIC HEALTH IMMUNIZATION PRACTICES. *Am J Epidemiol.* 1971;94(3):179–189. 10.1093/oxfordjournals.aje.a121310 5093648

[ref-10] Gavi: Facts and figures: Statistics measuring our impact on global immunization.2020. Reference Source

[ref-11] Jain P Humienny R: Normative influences on the role of prescription medicine misuse among college students in the United States. *Health Commun.* 2020;35(3):331–340. 10.1080/10410236.2018.1563029 30628463

[ref-12] Jang SA Rimal RN Cho N: Normative influences and alcohol consumption: The role of drinking refusal self-efficacy. *Health Commun.* 2013;28(5):443–451. 10.1080/10410236.2012.691455 22809467

[ref-13] JHPIEGO: Site assessment tool for inpatient postpartum care basic information. Part A: Equipment and supplies. In A. Blouse, P. Gomez, & B. Kinzie (Eds.), *Site assessment and strengthening for maternal and newborn health programs.*JHPIEGO.2004;1–6.

[ref-70] Johnson KF: The influence of school connectedness and academic self efficacy on self-reported norm related pro-social behavior.Doctoral dissertation. Pennsylvania State University.2011. Reference Source

[ref-14] Johnson E Shu SB Dellaert BGC: Beyond nudges: Tools of a choice architecture. *Market Lett.* 2012;23:487–504. 10.1007/s11002-012-9186-1

[ref-15] Lapinski MK Anderson J Shugart A: Social influence in child care centers: A test of the theory of normative social behavior. *Health Commun.* 2014;29(3):219–232. 10.1080/10410236.2012.738322 23682754

[ref-16] Matsumura T Nakayama T Okamoto S: Measles vaccine coverage and factors related to uncompleted vaccination among 18-month-old and 36-month-old children in Kyoto, Japan. *BMC Public Health.* 2005;5(1):59. 10.1186/1471-2458-5-59 15935101 PMC1177963

[ref-17] Ministry of Health and Population Nepal: National immunization programme. Government of Nepal.2020. Reference Source

[ref-18] Ministry of Health Nepal: Nepal Health Sector Strategy Implementation Plan 2016-2021. Government of Nepal.2017. Reference Source

[ref-19] Ministry of Health Nepal Department of Health Services: Annual Report 2016-17.2018. Reference Source

[ref-20] Ministry of Health Nepal, New ERA, & ICF: Nepal Demographic and Health Survey 2016.2017. Reference Source

[ref-21] Nandi A Shet A: Why vaccines matter: Understanding the broader health, economic, and child development benefits of routine vaccination. *Hum Vaccin Immunother.* 2020;1–5. 10.1080/21645515.2019.1708669 31977283 PMC7482790

[ref-22] National Planning Commission: Nepal’s sustainable development goals, baseline report.Government of Nepal. 2017. Reference Source

[ref-23] Njau JD Cairns LK: A literature review on the economic benefits of vaccines in low and middle income countries: Evaluating progress in the era of ‘ *a decade of vaccines*’ initiative. *Vaccine Reports.* 2016;6:62–76. Scopus. 10.1016/j.vacrep.2016.11.002

[ref-24] Odusanya OO Alufohai EF Meurice FP: Determinants of vaccination coverage in rural Nigeria. *BMC Public Health.* 2008;8(1):381. 10.1186/1471-2458-8-381 18986544 PMC2587468

[ref-25] Owino LO Irimu G Olenja J: Factors influencing immunisation coverage in Mathare Valley, Nairobi. *East Afr Med J.* 2009;86(7):323–329. 10.4314/eamj.v86i7.54146 20499781

[ref-26] Ozawa S Clark S Portnoy A: Estimated economic impact of vaccinations in 73 low- and middle-income countries, 2001-2020. *Bull World Health Organ.* 2017;95(9):629–638. 10.2471/BLT.16.178475 28867843 PMC5578376

[ref-27] Ozawa S Mirelman A Stack ML: Cost-effectiveness and economic benefits of vaccines in low- and middle-income countries: A systematic review. *Vaccine.* 2012;31(1):96–108. 10.1016/j.vaccine.2012.10.103 23142307

[ref-28] Paul A: Rejoice Architecture Meets Social Norms to Accelerate Vaccination in Nepal: Protocol for a Mixed-Method Quasi-Experimental Study. 2020. 10.17605/OSF.IO/JYBK4 PMC8028845 33870101

[ref-29] Perry H Nurani S Quaiyum M: Barriers to immunization among women and children living in slums of zone 3 of Dhaka City, Bangladesh: A qualitative assessment. *icddr,b Knowledge for Global Lifesaving Solutions*. 2020. Reference Source

[ref-30] Rainey JJ Watkins M Ryman TK: Reasons related to non-vaccination and under-vaccination of children in low and middle income countries: Findings from a systematic review of the published literature, 1999 – 2009. *Vaccine.* 2011;29(46):8215–8221. 10.1016/j.vaccine.2011.08.096 21893149

[ref-31] Rimal RN: Modeling the relationship between descriptive norms and behaviors: A test and extension of the theory of normative social behavior (TNSB). *Health Commun.* 2008;23(2):103–116. 10.1080/10410230801967791 18443998

[ref-32] Rimal RN Real K: How behaviors are influenced by perceived norms: A test of the theory of normative social behavior. *Communication Research.* 2005;32(3):389–414. 10.1177/0093650205275385

[ref-33] Scholz S Ngoli B Flessa S: Rapid assessment of infrastructure of primary health care facilities – a relevant instrument for health care systems management. *BMC Health Serv Res.* 2015;15:183. 10.1186/s12913-015-0838-8 25928252 PMC4421986

[ref-34] Shrestha S Shrestha M Wagle RR: Predictors of incompletion of immunization among children residing in the slums of Kathmandu valley, Nepal: A case-control study. *BMC Public Health.* 2016;16(1):970. 10.1186/s12889-016-3651-3 27619349 PMC5020516

[ref-35] Thaler RH Sunstein CR: Nudge: Improving decisions about health, wealth, and happiness.New Haven: Yale University Press. 2008. Reference Source

[ref-36] Thaler RH Sunstein CR Balz JP: Choice architecture.In Shafir E (Ed.), *The behavior foundations of public policy.*Princeton University Press.2014;428–439. 10.2139/ssrn.2536504

[ref-37] Vonasek BJ Bajunirwe F Jacobson LE: Do maternal knowledge and attitudes towards childhood immunizations in rural Uganda correlate with complete childhood vaccination? *PLoS One.* 2016;11(2):e0150131. 10.1371/journal.pone.0150131 26918890 PMC4769080

[ref-38] The World Medical Association: Communicable diseases: Diseases transmitted by bacteria and viruses. 2020. Reference Source

[ref-39] Wilson JQ Kelling GL: Broken windows.Atlantic Monthly.1982;29–36. Reference Source

[ref-40] World Health Organization: Immunization coverage: Key facts. 2019. Reference Source

