# Supplementary material for: Rejoice architecture meets social norms to accelerate vaccination in Nepal: Protocol for a mixed-method quasi-experimental study
Source: Gates Open Res. 2021 Sep 22;4:121. Originally published 2020 Aug 26. [Version 2] doi: 10.12688/gatesopenres.13168.2 (PMC8028845; doi:10.12688/gatesopenres.13168.2)
Supplement: Peer review history

# Peer review: Reviewer response for version 1

**DOI:** 10.21956/gatesopenres.14367.r30495

**Published:** 2021-04-06

**Authors:** Robert A. Bednarczyk

**Affiliations:** 1 Rollins School of Public Health, Emory University, Atlanta, GA, USA

This protocol describes the Rejoice Architecture intervention, designed to improve childhood vaccination in Makwanpur, Nepal. This mixed-methods study aims to utilize three theories - choice architecture, broken windows, and normative social behavior - to guide development, implementation, and evaluation of the intervention. 

 There are some clarifications needed to help this manuscript:

1. In the second paragraph of the introduction, please indicate which ages are under evaluation when describing 20 million children who are not appropriately vaccinated. Additionally, what percentage of the global childhood population does this represent (either in total or in LMIC)?
2. In the first paragraph under "National Immunization Programme", please spell out rotavirus rather than saying "rota". Additionally, the language here is difficult to follow because in some cases it seems like a disease is presented and in some cases the specific vaccine is presented. Please edit for clarity (e.g. "vaccines against seven additional diseases...").
3. I am a bit concerned about the use of Broken Windows theory, as there have been some studies that have shown that this is a flawed theory, specifically as it relates to crime in the US.  Has this been used to guide interventions (vaccine-related or in other settings) in Nepal?  
4. In the Methods, you refer to the geographic location of Makwanpur as maximizing "external validity" but given differences across Nepal, as indicated by highlighting the Terai and mountain regions, I am unclear by what you are referring to. This is especially of concern when, in the limitations, you reference generalizability issues given the location of the intervention.
5. Can you reference the population of Makwanpur, both in terms of numbers and breakdown of other characteristics relative to the general Nepal population, to help address some of these generalizability issues. 
6. Why are you doing IDI for fathers/grandmothers and FGD for mothers? 
7. In the sampling section of the Quantitative Methods, you describe sample size estimation with a starting point of 83% vaccinated, but this contradicts the 77% referenced in the first paragraph of the Methods.
8. In the analysis of the qualitative methods, you talk about recordings, but don't directly discuss how this recording will be done, how files will be stored, etc. 
9. A figure describing the timeline for the project, to clarify the evaluation period, timing of surveys, etc. would be helpful. 

Is the study design appropriate for the research question?

Yes

Is the rationale for, and objectives of, the study clearly described?

Yes

Are sufficient details of the methods provided to allow replication by others?

Yes

Are the datasets clearly presented in a useable and accessible format?

Not applicable

Reviewer Expertise:

Infectious disease epidemiology, vaccine acceptance, global health

I confirm that I have read this submission and believe that I have an appropriate level of expertise to confirm that it is of an acceptable scientific standard, however I have significant reservations, as outlined above.

# Peer review: Reviewer response for version 1

**DOI:** 10.21956/gatesopenres.14367.r30498

**Published:** 2021-03-24

**Authors:** Nimesh Poudyal, Ondari D. Mogeni

**Affiliations:** 1 Department of Epidemiology and Public Health Research, International Vaccine Institute, Seoul, South Korea; 2 Department of Interventions and Implementation Research, International Vaccine Institute, Seoul, South Korea

The article describes a well-designed study protocol to develop an intervention termed "Rejoice Architecture" within the conceptual framework of choice architecture, broken windows theory, and theory of normative social behavior and evaluate the impact of the intervention on immunization behavior and intentions through mixed-method quasi-experimental study. The rationale on the need for developing such an intervention and approach for evaluation of the impact is well laid-down by the authors and the study objectives are also clearly described in the protocol. It is clear that the study aims to understand the challenges of immunization uptake and measuring the impact of intervention instituted.

 The overall design of the study is replicable, but it would be good if the authors are able to describe the study setting further elaborating the population demographics and in particular under 2 years and how this compares to the rest of the country, the size of Makwanpur district, and what is the mortality per 1000 live births, in order to put the vaccine coverage of 76.7% into perspective. Additionally, it may be good to add a justification for the selection of that particular study area and describing the selection criteria used. Furthermore, of the units to be included in the study, some will be categorized into "treatment group" and "control group". Providing more details on what qualifies a unit to be termed "control group" and providing specific activities/intervention planned in each group will help understand the study design more clearly for its replication. One ‘control group’ is ultimately excluded during the intervention stage of the study and it is not clear what role this excluded group serves.

 It may also be good to consider including a section on data management. The study design in the article mentions the use of handheld tablets, but it does not provide any detail on how the data will be managed throughout the study period, where it will be warehoused, how data safety and security will be ensured, who will be able to access the data? Is there an egalitarian approach given this is a collaboration?

 One must be 18 years and over to be eligible to participate in the study. Given the challenges faced by mothers who are less than 18 years old in accessing immunization services, not including them during the formative assessment may lead to a bias on the designing of the intervention. A few articles seem to suggest teenage motherhood is prevalent in the area as well as in the country:

1. https://kathmandupost.com/national/2018/02/15/teenage-pregnancy-rife-in-makwanpur
2. http://www.bibalex.org/Search4Dev/files/428878/455706.pdf

It is not clear in the consenting process whether it will be done individually or collectively in the case of focus group discussion. And will each participant sign an informed consent form? Will a signed copy be extended to them?

 It is mentioned that the respective IRBs have approved the study. It will be helpful to include the IRB approval number from respective IRBs for future reference. 

 It is great that part of the dissemination process includes engagements of the participating Palikas. Will this be done at the community level or at the official level? Though it is mentioned that the results may not be generalized, it may be good to provide this feedback to stakeholders such as government officials to consider the study at a larger scale more so if the results are positive and given the overall goal is to increase immunization coverage.

 There is mention of COVID-19 and its impact. What may be the mitigation measures if travel is not possible?

Is the study design appropriate for the research question?

Yes

Is the rationale for, and objectives of, the study clearly described?

Yes

Are sufficient details of the methods provided to allow replication by others?

Partly

Are the datasets clearly presented in a useable and accessible format?

Not applicable

Reviewer Expertise:

Vaccinology, Infectious Disease, Epidemiology

We confirm that we have read this submission and believe that we have an appropriate level of expertise to confirm that it is of an acceptable scientific standard.

# Peer review: Reviewer response for version 1

**DOI:** 10.21956/gatesopenres.14367.r30492

**Published:** 2021-03-22

**Authors:** Ann M. Weber

**Affiliations:** 1 School of Community Health Sciences, University of Nevada, Reno, NV, USA

This is a well-written manuscript of an ambitious study to improve childhood immunization rates in Nepal. Overall, the protocol is clear and easy to follow. The introduction very nicely lays out the importance of the issue, the local context, and the facilitators and barriers to increased uptake. The open source links to the qualitative interview guides and surveys are particularly appreciated and very helpful. My major comments are for 1) consideration of addressing the normative behavior aspects of the issue in more depth, 2) recommendations to describe plans for the intervention itself in more detail, and 3) improved clarity for the justification of some of the study design decisions.

 Major comments:

 1. The inclusion of fathers and grandmothers is an important component of the qualitative research and understanding normative influences around the barriers to immunization of children. It might be helpful to readers to briefly explain the reasoning behind their inclusion. Importantly, transforming social norms is difficult, but possible. It requires engaging stakeholders at multiple levels (including fathers, grandparents, village leaders) and across multiple domains (of families, communities, and institutions) in the intervention. It would be helpful to understand how various stakeholders are involved in the intervention itself as well as in the quantitative aspect of the study, if at all. For example, are fathers and grandmothers engaged as part of the intervention? Will they be interviewed or surveyed after the program implementation to assess change in their beliefs, knowledge or attitudes? If no to both questions, I would recommend that the researchers consider opportunities to include them throughout the study process.

 2. As a reader, I would appreciate more detail on the plans for the intervention itself (in addition to the question above about fathers and grandparents) that address some of the barriers raised in the introduction. For example, will health workers be trained to address barriers related to how families are treated in the clinics (assuming this issue if raised in the qualitative portion)? The implementation of an appointment reminder system seems to be a key element to addressing the essential enabler that: “parents know and remember this schedule in order to bring their children to the facility at the appropriate time.” How might such an appointment system be implemented, for example, with text messages to moms?  Does it require an electronic records system to be implemented in the clinics? How does the intervention address the issue of loss of immunization cards?

 3. There are some elements of the study design that aren’t clearly justified or motivated for the reader. For example:

- The justification of non-random selection of wards and clinics within wards is unclear and I would encourage the researchers to consider the possibility of performing a cluster randomized study. The researchers state that they want to “maximize geographic diversity across the three Palikas.”  However, this might be accomplished with stratified sampling.  Athey and Imbens recommend “researchers to stratify the population into small strata and then randomize within the strata and adjust the standard errors to capture the gains from the stratification” and furthermore “that one should always stratify as much as possible, up to the point that each stratum contains at least two treated and two control units.” (Athey and Imbens, 2017 ^1^).
- Is limiting the participants to a 1 km radius of a study health facility for ease of access for the research? If so, are the researchers concerned that they might be overlooking an important barrier to immunization uptake due to the time cost of travel in terms of generalizability to participants living further away? How are they trying to address this concern?
- Why are mothers only part of focus groups and not planned for any in-depth interviews? It might be useful to ask mothers in-depth questions about the people in her life that influence her behavior and the same questions about household dynamics that are asked of fathers and grandmothers.
- The checklist evaluation of the intervention implementation may miss opportunities to better understand implementation challenges. It might be valuable to have more quantitative data to understand implementation or process issues not picked up by the checklist in the event that the program is not as effective as hoped. For example, will any of the key health workers by surveyed before and after the intervention? How will the appointment system be evaluated?

Minor comments:

- Methods - Second paragraph – “For each ward in the treatment arm, we selected another ward and clinic as a *treatment* site in that Palika, matched on population characteristics with the treatment ward.” Did the authors mean to write “…we selected another ward and clinic as a *control* site in that Palika…”?  Also, what are the population characteristics that they will match on?
- Qualitative - What is the rationale for excluding one control site from the collection of qualitative data?
- Quantitative - The ICC of 0.01 seems very small. How was this value determined?
- Will administrative data be available on overall vaccination rates in the communities?
- A 20% attrition rate after 8 months seems high. Why do the researchers think that there will be so much attrition? Is there any action that could be ethically included in the study to minimize attrition?

Is the study design appropriate for the research question?

Yes

Is the rationale for, and objectives of, the study clearly described?

Yes

Are sufficient details of the methods provided to allow replication by others?

Partly

Are the datasets clearly presented in a useable and accessible format?

Not applicable

Reviewer Expertise:

Child health and development; program impact evaluations in low- and middle-income countries; gender inequality and norms; health disparities.

I confirm that I have read this submission and believe that I have an appropriate level of expertise to confirm that it is of an acceptable scientific standard, however I have significant reservations, as outlined above.

## References

[1] : The Econometrics of Randomized Experiments a.1: 10.1016/bs.hefe.2016.10.00373-14010.1016/bs.hefe.2016.10.003
